# Measuring emotional and social wellbeing in Aboriginal and Torres Strait Islander populations: an analysis of a Negative Life Events Scale

**DOI:** 10.1186/1475-9276-6-18

**Published:** 2007-11-14

**Authors:** Emma Kowal, Wendy Gunthorpe, Ross S Bailie

**Affiliations:** 1 Menzies School of Health Research, Institute of Advanced Studies, Charles Darwin University, Darwin, Australia; 2 School of Social and Environmental Enquiry, University of Melbourne, Victoria, Australia

## Abstract

Aboriginal and Torres Strait Islander Australians experience widespread socioeconomic disadvantage and health inequality. In an attempt to make Indigenous health research more culturally-appropriate, Aboriginal and Torres Strait Islander Australians have called for more attention to the concept of emotional and social wellbeing (ESWB). Although it has been widely recognised that ESWB is of crucial importance to the health of Aboriginal and Torres Strait Islander peoples, there is little consensus on how to measure in Indigenous populations, hampering efforts to better understand and improve the psychosocial determinants of health. This paper explores the policy and political context to this situation, and suggests ways to move forward. The second part of the paper explores how scales can be evaluated in a health research setting, including assessments of endorsement, discrimination, internal and external reliability.

We then evaluate the use of a measure of stressful life events, the Negative Life Events Scale (NLES), in two samples of Aboriginal people living in remote communities in the Northern Territory of Australia. We argue that the Negative Life Events Scale is a promising assessment of psychosocial wellbeing in Aboriginal and Torres Strait Islander populations. Evaluation of the scale and its performance in other samples of Aboriginal and Torres Strait Islander populations is imperative if we hope to develop *better*, rather than *more*, scales for measuring ESWB among Indigenous Australians. Only then will it be possible to establish standardized methods of measuring ESWB and develop a body of comparable literature that can guide both a better understanding of ESWB, and evaluation of interventions designed to improve the psychosocial health of Indigenous populations and decrease health inequalities.

## Background: Policy advancements, methodological problems

The poor health of Australia's Indigenous people relative to the nation's population is well documented. Indigenous people on average live 17 years less than other Australians, and suffer higher rates of nearly every type of illness and injury [1,2]. The reasons for these disparities are complex, but undoubtedly relate to a history of colonisation and ongoing disadvantage. Australia's approximately 450,000 Indigenous people, which make up 2.4% of the population, are more likely to live in remote areas far from services, less likely to be educated or employed, more likely to be jailed, and less likely to have adequate housing [2].

While the well-known determinants of health inequality, such as employment, education and wealth, have been extensively studied in the Aboriginal and Torres Strait Islander population, it is only recently that the social and emotional aspects of health have received much attention. Since the 1990s it has been widely recognised in Australia that mental illness and stress are significant problems for Indigenous people, as well as direct causes, moderators and modifiers of physical ill-health [3-5].

The increase in interest in this area has been in response to the efforts of Indigenous leaders to raise the profile of mental health/Emotional and Social Wellbeing (ESWB) on the national policy agenda, through what has been called the Indigenous Mental Health Movement [[6]:85] (Note that the term 'Emotional and Social Wellbeing' is currently the term used within Aboriginal and Torres Strait Islander health policy to represent an area that includes mental health [7]). It represents, in part, an attempt to recognise Indigenous definitions of health which see health holistically [[8]:ix]. Indigenous people have expressed that ESWB is important for its own sake and have outlined some of its dimensions:

*Enhancing emotional and social wellbeing involves support for healthy relationships between families, communities, land, sea and spirit...A focus on strengthening communities and culture is fundamental to empowering individuals and communities to identify and meet their own needs. Strong healthy communities are those where individuals experience a sense of belonging, trust, participation and social support* [[9]:9].

Elsewhere, Indigenous people have argued that ESWB is crucial for good physical health:

*Aboriginal people emphasised the strong relationship of mental health and well-being to physical health and saw loss of mental well-being as contributing in a major way to the poor physical health and health outcomes of Aboriginal people. There is much to suggest that this is indeed a further significant and major contributor to the adverse and deteriorating state of the health of Aboriginal people* [[3]:1]

This view is congruent with models of the social determinants of health deriving from the field of social epidemiology. A prominent model sees 'upstream' factors such as housing policy and educational attainment influencing 'midstream' factors such as psychosocial factors, health behaviours and access to health care, which in turn influence 'downstream' factors, in this case physical health. Concepts of ESWB fit most clearly as psychosocial factors in this model, alongside stress, control, depression, self-esteem, coping, and anger [[10]:436]. It is also recognised that mental health issues are responsible for a significant proportion of morbidity worldwide [11].

It would seem, then, that both Indigenous and Western perspectives see ESWB as critical in understanding and addressing health inequalities. The Australian Government has responded to this by developing national policies for Indigenous Emotional and Social Wellbeing and funding numerous programs including the establishment of 16 Emotional and Social Wellbeing regional centres within community-controlled Aboriginal and Torres Strait Islander health services, where Indigenous people can access free culturally-appropriate counseling [7,12,13].

Unfortunately, these significant policy and program innovations have not been matched by relevant, quality population research. The research effort into Indigenous concepts of ESWB and its links to mental and physical health have been hampered in part by a lack of validated scales for measuring ESWB in Indigenous populations. Below we address some reasons behind this tardiness of methodological development despite the prominence given to ESWB in Indigenous health policy.

First, the appropriateness of Western concepts for representing Indigenous concepts has been questioned. Scholars in the emerging field of Indigenous psychology argue that "psychological theories reflect the values, goals and issues of the United States of America and they are not generalisable to other societies," and that "psychological theories have been used and continue to be used as a tool for cultural dominance [[14]:76]." In Australia, a similar discourse exists, arguing that the presumption of universality and a preoccupation with individualism are the core reasons why Western psychological concepts are inappropriate and potentially damaging to Indigenous people [6]. The Australian Psychological Society currently advises caution with using psychological scales with Aboriginal and Torres Strait Islanders as "there are currently no known formal psychological tests that have been developed specifically for use with indigenous people and that provide current-day norms and measurement statistics for indigenous test takers [[15]:3]." Similarly, Australia's National Health and Medical Research Council cautions researchers to take heed of the many cultural differences between Indigenous and non-Indigenous people, as well as differences between Indigenous peoples [[16]:3]. Indigenous and non-Indigenous scholars in Canada, New Zealand and the United States have expressed similar sentiments [17-20].

To date, only one tool has been developed specifically for measuring mental health status in Australian Indigenous youth populations, and the initial validation of this tool has not yet been replicated [21]. While this advance is to be applauded, scales to measure other aspects of ESWB are urgently required. Outside Australia, there has been further progress in the development of scales specifically for the measurement of mental health status in Indigenous people. Important examples include the *Hua Oranga *scale developed in New Zealand to measure mental health outcomes in Maori populations [22], and the *Voices of Indian Teens *survey developed to measure aspects of mental health and substance misuse in American Indian adolescents [23]. The large *Circles of Care *program in native communities in the United States found that most communities developed their own scales to evaluate the process and quality of mental health care, while others used mainstream scales [24].

Second, even if it could be successfully argued that it is appropriate to apply Western psychological concepts such as stress, self-efficacy and depression to Indigenous people, there is no consensus on how these concepts could be measured in individuals and populations. It has been suggested that an in-depth psychological interview involving cultural consultants is the most appropriate way of assessing the mental health and ESWB of individual Indigenous people, and that this is the most appropriate method for research [21,25]. As this method requires a significant commitment of time and resources, it is not usually practicable for research projects involving large numbers of people. In order to develop an understanding of ESWB within populations, rather than just individuals, scales must be available that are effective and acceptable to these populations.

This is also necessary as health researchers, increasingly interested in the links between mental and physical health, are increasingly using mainstream psychological scales in their health research projects. In other Indigenous populations, such as Native Americans in the United States, many mainstream scales have been evaluated (for example [26,27]. More recent research can use these mainstream scales with some confidence that they are valid in the Native American context [28,29]. In Australia, where this earlier research effort has not been replicated, the use of mainstream scales with Indigenous populations is on shakier ground. Where mainstream scales are used by health researchers in Australia, it is common that correlations of the scales with health outcomes are simply reported, without a comprehensive evaluation of the scales' performance [30]. Health researchers are then forced to try and explain correlations they have found without knowing whether the scale effectively measured what it was intended to measure. It is important for this group of researchers that consensus be reached on a set of psychological scales that are appropriate and valid for use in Australian Indigenous populations.

Some Australian health researchers have taken heed of these warnings and developed novel scales for a particular Indigenous community [31-33]. Often, the resourcing of the project and/or the methodology mean the scale is not adequately evaluated. In other cases, the results are not published in journals and are thus difficult to access. Westerman argues that in this area of research, the belief that " 'what works for one Aboriginal group will not be valid for another'...is perhaps the greatest obstacle to the provision of conceptually and empirically sound research in this area of enquiry" [21].

Thus there are two poles of opinion regarding the use of Western scales with Indigenous populations: one using mainstream scales; and the other, developing new scales for each community. Both extremes contribute to the lack of sound published data, and the resulting lack of consensus among the research community on valid scales for measuring ESWB across Indigenous populations. The lack of quality published data also means that researchers cannot draw on the work of others, perpetuating a cycle of over-research and potentially poor quality research.

The research community has a responsibility to ensure that health inequalities are addressed in a manner acceptable to Indigenous people, and that Indigenous people are not exposed to poor quality research or over-researched. In order to fulfill those responsibilities, it is imperative that the Australian research community attempt to generate some consensus about the best scales to use to measure ESWB, so that the body of evidence in this area increases, and the chance of Indigenous people benefiting from their participation in research is maximized.

This paper attempts to take a step toward consensus on scales for measuring ESWB by reporting on the use of a Negative Life Events Scale (henceforth NLES) to measure stress in two samples of Indigenous Australians. We also outline the basic methodological principles for establishing the validity of scales, intended to provide guidance for health researchers who wish to evaluate scales used in their research.

## The Negative Life Events Scale (NLES)

Measures of negative life events are one method of measuring exposure to stress. Stress is a multivalent term used to refer to the experience of adverse stimulation, the source of the adverse stimulation, and/or the organism's response to the adverse stimulation [34]. Stress has been linked to a variety of adverse mental and physical health outcomes in many populations over the last decades [35]. Measuring stressful life events is one way of measuring exposure to stress, the others being 'chronic stressors' and 'daily hassles and uplifts' [36]. International work has found that stress is a salient construct in many non-Western nations [37-40].

From an Indigenous perspective, stress is one aspect of ESWB. Stress has been reported as a salient term for Indigenous Australians and other Indigenous peoples [41-43], and as a key factor impacting on health, for instance on the success of smoking cessation [44,45]. In particular, in discussing the Stolen Generations, the 20^th ^century policies of removing children of mixed race from their Indigenous parents and placing them in institutions, Indigenous Australians frequently draw on notions of trauma and stress to understand their experiences and their effects [46].

The Australian Bureau of Statistics has used a Negative Life Events Scale (NLES) to measure chronic stress in the national Indigenous population. The particular items in the scale were chosen by the Bureau after extensive consultation with national Indigenous representatives [47]. Details of the process of consultation employed by the Bureau have not been published, but included consultation with Indigenous mental health experts in the Indigenous community-controlled health sector, and extensive pilot-testing of the scale to ensure face validity. The use of this scale in 2002 indicated that Indigenous Australians experience more stressful life events than non-Indigenous Australians. Of those surveyed, 82% of Indigenous people but only 57% of non-Indigenous people reported experiencing one or more stressful life event in the previous year [[47]:5].

While the NLES has been used in this national setting, psychometric analysis of the scale has not yet been performed. This paper evaluates the use of the NLES in a smaller study of Indigenous people in eleven communities in the Northern Territory. Table 1 outlines the version used in this study.

**Table 1:** Negative Life Events Scale.

**Have any of these things been a worry for you or anyone else living in this house during the last year?**
Serious illness
Serious accident
Death of family member or close friend
Divorce or separation
Not able to get a job
Lost job
Alcohol related problems
Drug related problems
Seeing fights or people beaten up
Abuse or violent crime
Trouble with the police
Gambling problem
Member of family sent to jail
Overcrowding at home
Discrimination/Racism
Vandalism or Malicious damage to property

The scale is the most simple type of Life Events Scale, where respondents answer 'yes/no' to each item, only negative events are listed, and no weighting or subjective rating of event impact are included. The scale also underwent minor amendments for its use in the present study, as explained below.

This sample reported here was collected as part of the Housing Improvement and Child Health study which was funded in 2002 by Australia's National Health & Medical Research Council. The project is a partnership between the Menzies School of Health Research, the Indigenous Housing Authority of the Northern Territory (IHANT) and the participating remote Indigenous communities. The general aim of the study is to assess the impact of improved housing stock on the health of young children, and to understand the factors that may mediate this relationship. The study has been conducted in eleven remote communities in the Northern Territory, both in the tropical north and arid central Australia. All of these communities are small (ranging from 70 to 1500 people), isolated, and poorly-resourced. Most residents have an Indigenous first language, with English as a second, third or fourth language.

The aspect of data collection relevant to this paper were questionnaires delivered to heads of households (n = 262), henceforth called 'householder sample', and to carers of young children within those households (n = 373), henceforth called 'carer sample'. The NLES was contained within a longer questionnaire that included standard demographic data (income, education, work status), questions about house maintenance, links to traditional lands and financial stress. Householders and carers of children were recruited from houses where young children resided, which represented an average of 73% of the occupied houses in the communities. Thus this was not a representative sample of Indigenous adults from participating communities. However, it was a sample comprised of a diverse Indigenous population of many language groups, spread over a large geographical area.

As a result of consultation with Indigenous stakeholders, the wording of the question asked was changed. Instead of "In the last year, have any of these been a problem for you, or your family and friends?", participants were asked, "Have any of these things been a worry for you or anyone else living in this house during the last year?". The change from "problem" to "worry" reflected the Aboriginal English in use in the Northern Territory. Indigenous advisors felt that the word "worry" was equivalent to "problem" but would be more widely understood. The other change was the use of the phrase "you or anyone else living in this house" instead of "you, or your family and friends". This reflected the focus of the larger study on the effect of housing on the social, emotional and physical health of residents. Indigenous advisors and genealogical data collected indicated that members of a single household were, in all cases, also family members. As we did not ask respondants about family and friends in other households, it is possible the change of wording led to an under-reporting of negative life events. The item "vandalism or malicious damage to property" was added, reflecting this particular project's interest in housing. In addition, the wording of some items was changed slightly for clarity in the local Aboriginal English without changing the meaning of the question. For example: 'Witness to violence' was changed to 'seeing fights, or seeing people beaten up'.

The process of adapting the NLES for use in this study was not ideal. Neither the Australian Bureau of Statistics nor this research team engaged a formal process of scale development through cultural validation which is the gold-standard method of scale construction or adaptation. Thus the scale developed may not be as appropriate or valid as possible. However, the process of consultation with Indigenous stakeholders undertaken for this study is likely to resemble the most common process undertaken by health researchers with limited time, resources and expertise in scale development. While ideally an expert in cross-cultural scale development should be involved with health research projects where ESWB scales are used, we advocate that at the very least, scales are properly assessed in line with the method outlined below. In this way, researchers can become aware of any deficiencies in the performance of the scales they use or adapt *before *they correlate scales with health outcomes and attempt to interpret the results.

## Methods

A sound evaluation of a scale will include an assessment of the scale in its entirety, as well as individual items making up the scale, supplemented by a critical review of the literature that concerns the use of the scale in particular settings [48,49]. As mentioned earlier, the NLES has been used previously in Australian Indigenous settings. Following the 2002 National Aboriginal and Torres Strait Islander Social Survey (henceforth NATSISS), the scale was included in the 2004–5 National Aboriginal and Torres Strait Islander Health Survey (NATSIHS). This means that the scale has successfully passed a number of preliminary tests including approval by the National Advisory Group on Aboriginal and Torres Strait Islander Health Information and Data (NAGATSIHID), extensive pilot-testing in situ, and inherent justification of the additional respondent load and taxpayer expense associated with its inclusion in the NATSIHS [50]. Another version of the Negative Life Events Scale was recently used in a large study of carers of Indigenous children in Western Australia, and the distribution of responses was reported. However, as that scale shares only 6 out of 14 items with the scale used in this study, the data is not appropriate for comparison [51].

However, beyond this there is no published documentation of the performance of the NLES in Australian Indigenous settings. Below we report on our evaluation of the NLES in relation to three criteria of usefulness: endorsement, discrimination, and reliability.

### Endorsement

The frequency of endorsement refers to the usefulness of the scale to provide meaningful information about the target population. Endorsement of questions highlights the extent of missing data and potential problems with the question. Endorsement of response options for each question indicates whether each question, and the scale as a whole, effectively teases out differences in the target population. If the proportion of the sample who responds in the same way to a particular question is greater than 0.80 (or less than 0.20) then we need to question the usefulness of that question since we can predict what the answer will be with greater than 80% accuracy. In some circumstances, a high proportion of responses to a particular option can be expected, especially when 'nonsense' questions are included to detect biases, or if there is a 'yea-saying bias' [52] among the sample. Evidence of a 'yea-saying bias' may reflect a tendency for some ethnic minorities to affirm statements or questions to appease the questioner, rather than admitting they don't understand a question or don't know the answer [49,53]. Endorsement rates were calculated for each item in the NLES. Based on recommendations by Streiner and Norman [49], items with endorsement rates between 0.20 and 0.80 were considered acceptable.

### Discrimination

Discriminative ability of items in the NLES indicates the usefulness of each item in terms of its contribution to the scale overall. A discriminative index, calculated for each item in the NLES tells us how well the item discriminates between those individuals who score high on the scale overall and those who score low. We would expect that those respondents who have a high score on the whole scale would also have a high probability of answering 'yes' to any given item on the scale. A low discriminative index suggests that the item offers little information on which to base assumptions about the relationship between the item and the intended purpose of the scale overall (ie. as a measure of stress). The discriminative index (d) of each item (i) in the NLES was calculated using the formula:

d_i _= (U_i _- L_i_)/n_i_

where U_i _is the number of people above the median total score who answer positively to item i, L_i _is the number of people below the median total score who answer positively on item i, and n_i _is the number of people above the median total score [49]. Although closely related to endorsement, the discriminative index looks at each item in relation to all other items in the scale, rather than in isolation. That is, an item may show poor endorsement rates (less than 20%), but reasonably good discriminative ability if all persons endorsing the item were above the median total NLES score. Discriminative ability of items was assessed in both the Carer and Householder samples.

Ebel and Frisbie [54] provide the following rule of thumb for determining the usefulness of items in terms of their discriminative index: > 0.39 Retain items; 0.30–0.39 Possibilities for improvement; 0.2–0.29 Need to check and review items; 0.00–0.20 Discard or do in-depth review of items; < 0.00 Discard items.

### Reliability

Reliability of a scale refers to its ability to measure something consistently. There are two types of reliability: internal and external. Internal reliability of a scale refers to the extent to which the scale is consistent within itself, or whether the scale's items consistently measure different aspects of the same attribute. The most commonly used measure of internal consistency is Cronbach's alpha, or in the case of dichotomous data, the Kuder-Richardson formula 20 (KR-20) [[49,55]:248–292]. Nunnally [56] recommends that alpha should be above 0.70, but probably not higher than 0.90.

External reliability of a scale refers to the extent to which the scale varies from one use to another. One of the most common methods to assess the external reliability of a scale involves retesting the same people using the same scale, after a period of time has passed. However, the retest method can be problematic in the setting of remote community research. The conditions of data collection in remote communities make it difficult to achieve the ideal time delay of around a week between testing and retesting [55]. In addition, research participants are often reluctant to participate in retesting, perceiving that the researcher did not listen to them the first time or did not believe them [57].

Consequently, another method of assessing reliability was used – one that involved comparing the responses of married couples who lived in the same house, and had been living there for longer than one year. They were expected to show high agreement in their responses to the question: "Have any of these things been a worry for you or anyone else living in this house during the last year?" The Kappa statistic was used to assess the agreement between the responses of married couples.

There are no absolute cut-off points for Kappa coefficients. According to Fleiss [58], Kappa values in the range of 0.40 to 0.75 indicate fair agreement above chance and values exceeding 0.75 indicate strong agreement above chance. Landis and Koch [59] interpret Kappa values between 0.21 and 0.40 as indicative of fair agreement, values between 0.41 and 0.6 as indicative of moderate agreement, and values above 0.61 as indicative of substantial agreement. The cut-off for accepting a given Kappa coefficient will largely depend on the agreement that can be expected between two or more individuals. If the same individual was to complete the same scale on two separate occasions (true test-retest reliability), we might expect higher agreement than if two individuals completed the scale as was the case in this study. The guidelines of Landis and Koch [59] were used to interpret Kappa coefficients in this study. Data were extracted from both the Carer and Householder samples where one of each married couple was either a Carer or a Householder.

## Results

Table 2 compares the two samples in this study with characteristics of the national Indigenous population and the remote Indigenous population, of which the latter sub-population would be expected to more closely resemble the sample in this study. Overall, the samples in this study are more socioeconomically disadvantaged that the general or the remote Indigenous population. As one might expect, the Carer sample is largely female and younger than the householder sample. Although the Carer sample is less likely to be employed, their average income is higher than the Householder sample, reflecting the fact that many jobs on remote communities are low-paying, and Carers will often be in receipt of welfare payments to support the children they care for.

**Table 2:** Description of Samples

	**Carers**	**Householders**	Remote Indigenous*	All Aust Indigenous*
**n**	436	306		
**Median age**	27 (IQR: 22–35)	39 (IQR: 31–47)		
**% female**	91	48		
**Mean weekly income**	$313.24**	$274.57**	$350***	$387***
**% employed**	12.6	30	52	46
**%Completed year 10**	46	39	54	Not available
**%Reported at least one stressor on NLES**	96	96	85.5	82.3
**Mean number of stressors reported on NLES**	6.11 (sd = 3.65)	7.01 (sd = 3.68)	Not available	Not available

* Values from the Australian Bureau of Statistics [47]

** Individual income based on self-report

***Individual income based on mean equivalised gross household weekly income

Table 3 shows the endorsement of each item in the NLES in the two samples, as well as the rate of missing data. The endorsement rates from the 2002 NATSISS are included for comparison [47]. Three items showed endorsement rates of less than 20%: 'Divorce or separation' (15%), 'Not able to get a job' (12%) and Lost job' (3%). Two of these showed consistent rates in both the carers and householder samples in the HICH study as well as the remote-living sample in the NATSISS. The rate of missing data was less than 5% for all items in the Householder sample. In the Carer sample, the rate of missing data was less than 10% except for the item 'Vandalism or malicious damage to property' which had 16% missing data. The higher rates of endorsement for most items in these samples than in the NATSISS may reflect the lower socio-economic status of the sample.

**Table 3:** Endorsement of items in the Negative Life Event Scale

Item in the Negative Life Event Scale	Carers % responding 'yes' to item	% Missing data	Householders % responding 'yes' to item	% Missing data	NATSISS (remote only) % responding 'yes' to item
Serious illness	48	8	59	4	34
Serious accident	28	8	35	3	19
Death of family member or close friend	65	8	73	3	55
Divorce or separation	15	8	15	3	12
Not able to get a job	12	8	22	3	25
Lost job	3	9	7	4	5
Alcohol related problems	37	8	50	3	37
Drug related problems	29	8	43	3	n/a
Seeing fights or people beaten up	62	8	70	3	30
Abuse or violent crime	41	8	51	3	17
Trouble with the police	21	9	32	3	22
Gambling problem	36	9	41	4	26
Member of family sent to jail	32	8	35	3	25
Overcrowding at home	66	8	65	3	42
Discrimination/Racism	31	9	36	4	16
Vandalism or Malicious damage to property	34	16	49	4	n/a

**Figure 1:**
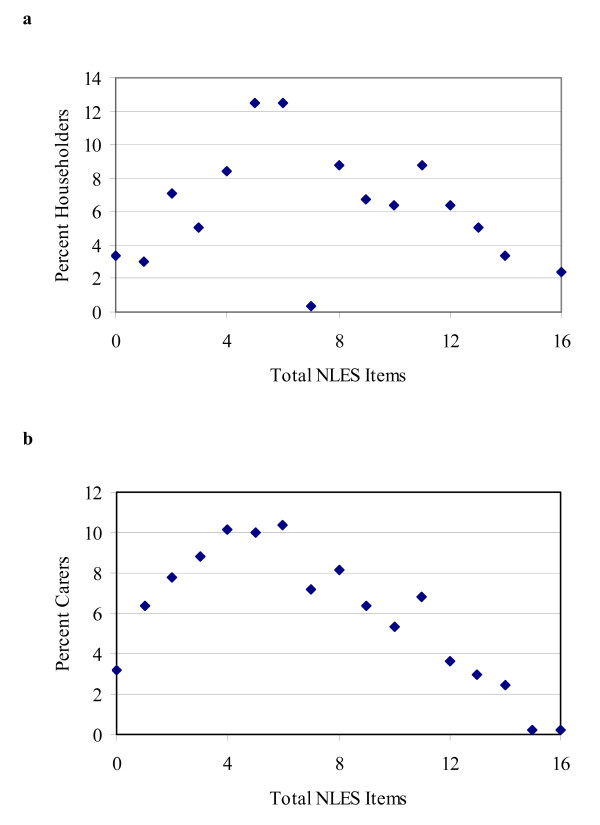
**Number of stressors reported in householder and carer samples**. a. Percentage of householders reporting total number of NLES items. b. Percentage of carers reporting total number of NLES items.

Figure 1 shows the percentage of respondents reporting each total number of stressors in the householder (1a) and carer (1b) samples. In both samples, there is a moderate peak at 4–6 stressors reported (10–12% of the sample), but overall there is a good spread of responses, also reflected by the standard deviation of both samples. The NLES is thus a suitable tool to distinguish between those more and less stressed within an Indigenous population.

Table 4 shows the discriminative index for each NLES item. In accordance with Ebel and Frisbie's [54] rule of thumb for determining the usefulness of items in relation to discriminative ability, there were two items of questionable usefulness: 'Not able to get a job' and 'Lost job'. These items discriminate less than 20% of the sub-sample who scored above the median NLES score. Two other items that warrant review are 'divorce or separation', and 'overcrowding at home' which discriminated 20–30% of those respondents who scored above the median NLES score. 'Death of family member or close friend' and 'serious illness' both showed good discriminative ability among the sample of carers, but not among the householder sample.

**Table 4:** Discriminative Index for each item of the NLES.

Negative Life Event	Carer Sample	Householder Sample
Serious illness	0.47	0.27
Serious accident	0.41	0.38
Death of family member or close friend	0.46	0.27
Divorce or separation	0.20	0.23
Not able to get a job	0.14	0.19
Lost job	0.06	0.09
Alcohol related problems	0.55	0.58
Drug related problems	0.51	0.55
Seeing fights or people beaten up	0.48	0.46
Abuse or violent crime	0.51	0.50
Trouble with the police	0.38	0.45
Gambling problem	0.42	0.47
Member of family sent to jail	0.35	0.33
Overcrowding at home	0.28	0.23
Discrimination/Racism	0.42	0.43
Vandalism or Malicious damage to property	0.52	0.67

Table 5 shows that the internal consistency of the NLES was high (0.80 and 0.81) among the carers sample and householder sample respectively. The alpha coefficients that correspond to the systematic elimination of each item indicate that no item threatens the internal consistency of the scale overall.

**Table 5:** Internal Consistency of items in the NLES

**NLES item removed**	**Carer sample α**	**Householder sample α**
Serious illness	0.80	0.80
Serious accident	0.80	0.79
Death of family member or close friend	0.81	0.79
Divorce or separation	0.81	0.79
Not able to get a job	0.81	0.80
Lost job	0.81	0.80
Alcohol related problems	0.79	0.78
Drug related problems	0.79	0.78
Seeing fights or people beaten up	0.79	0.78
Abuse or violent crime	0.79	0.78
Trouble with the police	0.79	0.78
Gambling problem	0.80	0.78
Member of family sent to jail	0.80	0.79
Overcrowding at home	0.79	0.80
Discrimination/Racism	0.80	0.78
Vandalism or Malicious damage to property	0.79	0.77
All negative life events included	**0.81**	**0.80**

Table 6 shows the agreement between responses of 65 married couples residing in the same household for one year or more, and who both completed the NLES (one as a 'householder', one as a 'carer'). Three items showed no statistically significant agreement: 'Divorce or separation', 'Not able to get a job' and 'Lost job'. Of the other items, approximately half the items showed fair agreement and half showed moderate agreement according to Landis and Koch's interpretation guidelines [59].

**Table 6:** Agreement between responses of married couples residing in same household for one year or more (n = 65).

Negative Life Event	Kappa statistic (95% confidence interval)
Serious illness	0.451 (0.238 – 0.663) *
Serious accident	0.529 (0.313 – 0.745) *
Death of family member or close friend	0.297 (0.053 – 0.541) *
Divorce or separation	0.200 (-0.134 – 0.533)
Not able to get a job	0.067 (-0.148 – 0.283)
Lost job	-0.026 (-0.076 – 0.025)
Alcohol related problems	0.355 (0.137 – 0.573) *
Drug related problems	0.459 (0.241 – 0.678) *
Seeing fights or people beaten up	0.535 (0.314 – 0.755) *
Abuse or violent crime	0.370 (0.142 – 0.598)*
Trouble with the police	0.275 (0.031 – 0.519)*
Gambling problem	0.525 (0.308 – 0.742)*
Member of family sent to jail	0.320 (0.089 – 0.550)*
Overcrowding at home	0.339 (0.114 – 0.564)*
Discrimination/Racism	0.247 (0.008 – 0.486)*
Vandalism or Malicious damage to property	0.539 (0.323 – 0.755)*

* Significant at p < 0.05

## Discussion

The evaluation of the NLES presented here indicates that most items in the scale perform well in relation to endorsement, discriminative ability and reliability. As this was a sample of a diverse group of Indigenous people spread over a wide geographical area, it is likely that all except three items in the NLES are appropriate for use in a wide spectrum of Indigenous communities.

Three items are of questionable usefulness to the scale. These items ask about divorce or separation, job loss, and unemployment. These three items were poorly endorsed by both samples, showed poor discriminative ability, and poor external reliability. These items did, however, show a high internal reliability, indicating the danger of only calculating an alpha score without other accompanying evaluative measures.

The poor endorsement rates for three items suggest that these phenomena are not widely experienced as stressors in this population. The poor discriminative ability of items about divorce or separation, job loss, and unemployment means that those respondents who did endorse these as being a problem for themselves or others living in the same house, were not among the more 'stressed' respondents in the sample. The poor agreement between married couples' responses to the NLES indicates that these items are not measuring what they are intended to measure, or are not measuring the phenomenon in a reliable fashion. For example, although 22% of householders reported 'not being able to get a job' was a problem for them or anyone else living in the house, it was unlikely that their spouse would report the same, despite both parties living in the same house. Analysis of responses by gender did not reveal a tendency for men or for women to answer in a particular way. This may suggest that these three items are ambiguous for the remote Indigenous respondent, and subsequently they are probably not a good measure of stress.

The reasons for the consistently poor performance of the three items in the NLES can only be ascertained by further research with this population, such as in-depth interviews with respondents and ethnographic research. One contributor may be the effect observed in underprivileged communities whereby social problems such as unemployment and relationship breakdown are so common as to become 'normalised', and thus not perceived as a cause of stress [60]. As this population was more socially disadvantaged than other Indigenous populations, one may predict that this effect is more pronounced than in other Indigenous populations. However, the Australia-wide remote Indigenous population surveyed in the NATSISS did report 'Not able to get job' slightly more frequently, but recorded poor endorsement of the other two items. Regardless of the explanations for the poor performance of the three items, though, it would be advisable to omit those items from the scale in future, leaving a 13-item scale.

The item 'overcrowding at home' was highly endorsed, but had a reasonably low discriminative index, suggesting that while many people experienced overcrowding, it was not a strong contributor to stress. This may be explained by over-reporting due to the setting of the study. As housing was a focus of the study, it is possible that research participants thought that if they reported that their house was overcrowded, they or their community may be more likely to receive resources for new houses or renovations.

The item 'vandalism and malicious damage to property', added to the NLES specifically for these samples, had a high proportion of missing data in one sample. This may relate to being the last item in the scale, or perhaps indicate that the language used in the item was inappropriate. However, the item was still well endorsed, and had a relatively high discriminative index and good external reliability. Those using the NLES in Indigenous populations should consider retaining it.

### Further research

The analyses described in this paper provide a useful overview of the NLES in terms of the usefulness of individual items making up the scale. Immediate further research could repeat these evaluative measures on samples where the NLES has already been used on Indigenous populations, such as the 2002 NATSISS and the 2004 National Aboriginal and Torres Strait Islander Health Survey [47,61]. This analysis would assess whether the NLES performs as well in a larger sample, and also confirm whether the three poorly-performing items should indeed be omitted from the scale. Further analyses are needed to evaluate the methods by which the NLES scale should be scored and interpreted as a measure of negative life stress. This would typically involve a factor analysis performed on the matrix of tetrahoric inter-item correlations, so as to be suitable for dichotomous data [62].

The finding that items relating to unemployment and to relationship breakdown were of questionable use invites further research into Indigenous people's perceptions of these events. More generally, the evaluation of the NLES presented here does not enable an assessment of construct validity, or the degree to which the NLES items accurately reflect the life events perceived as stressful for Indigenous people (note that although there has been research into the experiences of stress for Indigenous people [63,64], this body of research has not yet been integrated into the evaluation of scales suitable for population research). As well as qualitative research methods, this research effort could employ a culturally-competent clinical assessment of stress as a gold standard from which the construct validity of the scale could be assessed [21,21] [[65]:49–56].

When and if the NLES is established as a 'gold standard' measure of stress in Indigenous populations, it could serve as a tool for further research into the prevalence and experience of stress and related constructs for Indigenous Australians.

## Conclusion

Research into the links between emotional and social wellbeing and health inequalities will be greatly enhanced by health researchers routinely reporting on the evaluation of psychological scales they use, including endorsement, discrimination, and internal and external reliability. This will provide guidance as to which scales are performing well in diverse Indigenous populations, and assist in developing consensus in the field on a set of tools that are both effective and acceptable.

Ideally, a scale for measuring stressful life events would be developed wholly by Indigenous people for the Indigenous context. In the absence of such a scale, it is important to add value to the existing consultative frameworks employed by the Australian Bureau of Statistics and other researchers that adapt mainstreams scales for use in Indigenous populations. The NLES appears to generally perform well in the diverse Indigenous population sampled. The scale is a good starting point for further research into ESWB in Indigenous populations.

## Competing interests

The author(s) declare that they have no competing interests.

## Authors' contributions

EK conceived the research, drafted the article, assisted with data analysis and data collection. WG conceived the research, drafted parts of the article and conducted the data analysis. RB conceived and managed the larger study and reviewed the manuscript. All authors read and approved the final manuscript.
